# Isoform-specific effects of neuronal repression of the AMPK catalytic subunit on cognitive function in aged mice

**DOI:** 10.18632/aging.204554

**Published:** 2023-02-26

**Authors:** Xueyan Zhou, Wenzhong Yang, Xin Wang, Tao Ma

**Affiliations:** 1 Department of Internal Medicine-Gerontology and Geriatric Medicine, Wake Forest University School of Medicine, Winston-Salem, NC 27157, USA; 2 Department of Physiology and Pharmacology, Wake Forest University School of Medicine, Winston-Salem, NC 27157, USA; 3 Department of Neurobiology and Anatomy, Wake Forest University School of Medicine, Winston-Salem, NC 27157, USA

**Keywords:** AMPK, aging, protein synthesis, learning and memory, proteomics

## Abstract

AMP-activated protein kinase (AMPK) functions as a molecular sensor that plays a critical role in maintaining cellular energy homeostasis. Dysregulation of the AMPK signaling has been linked to synaptic failure and cognitive impairments. Our recent study demonstrates abnormally increased AMPK activity in the hippocampus of aged mice. The kinase catalytic subunit of AMPK exists in two isoforms α1 and α2, and their specific roles in aging-related cognitive deficits are unknown. Taking advantage of the unique transgenic mice (AMPKα1/α2 cKO) recently developed by our group, we investigated how isoform-specific suppression of the neuronal AMPKα may contribute to the regulation of cognitive and synaptic function associated with aging. We found that aging-related impairment of long-term object recognition memory was improved with suppression of AMPKα1 but not AMPKα2 isoform. Moreover, aging-related spatial memory deficits were unaltered with suppression of either AMPKα isoform. Biochemical experiments showed that the phosphorylation levels of the eukaryotic initiation factor 2 α subunit (eIF2α) were specifically decreased in the hippocampus of the AMPKα1 cKO mice. We further performed large-scale unbiased proteomics analysis and revealed identities of proteins whose expression is differentially regulated with AMPKα isoform suppression. These novel findings may provide insights into the roles of AMPK signaling pathway in cognitive aging.

## INTRODUCTION

Normal aging is associated with impairments of multiple aspects of cognitive function [1–4]. Dysregulation of brain energy metabolism has been linked to both the normal aging process and multiple neuronal disorders characterized by cognitive impairments such as Alzheimer’s disease (AD) [5–7]. AMP-activated protein kinase (AMPK) functions as a sensor at the molecular levels that plays a crucial role in regulation of cellular energy homeostasis [8]. Mounting evidence suggests a link between aberrant AMPK signaling and aging-related deficiencies of synaptic plasticity and dementia syndromes [9]. One important downstream effect of AMPK is the regulation of protein synthesis or mRNA translation, which is essential for the long-lasting form of memory and synaptic plasticity [10]. AMPK can regulate mRNA translation through suppression of the mammalian target of rapamycin complex 1 (mTORC1) or phosphorylation and inhibition of the eukaryotic elongation factor 2 (eEF2) by activating its kinase eEF2K [11]. We recently reported that AMPK signaling involves the phosphorylation of the α subunit of eukaryotic initiation factor 2 (eIF2α), which may serve as a key underlying molecular mechanism during memory formation [12, 13].

Mammalian AMPK is composed of catalytic α subunit, and β and γ subunits (regulatory). The kinase catalytic subunit has two isoforms α1 and α2 that, are encoded by separate genes (*Prkaa1* and *Prkaa2*) located in distinct chromosomes [14, 15]. AMPKα1 and α2 are both present in the brain, but whether and how regulation of different AMPKα isoforms is involved in neuronal function remain elusive. We recently reported, in young mice (3-6 months old), that neuronal repression of the AMPKα2 (not AMPKα1) results in cognitive defects and long-term synaptic plasticity impairments [12]. Meanwhile, we demonstrated abnormal hyperactive AMPK signaling in the hippocampus of aged mice [2]. In the current study, we aim to investigate how suppression of either AMPKα isoform in neurons may contribute to the regulation of cognitive and synaptic function associated with aging.

## MATERIALS AND METHODS

### Mice

Mice were housed in a barrier facility at Wake Forest School of Medicine. Operation of the facility complies with standards of the US Department of Agriculture’s Animal Welfare Information Center (AWIC), and the NIH Guide for Care and Use of Laboratory Animals. The facility is on a 12-hour light/dark cycle, with a regular cage-cleaning and feeding schedule. Female and male mice were both used. Polymerase chain reaction (PCR) was performed to verify the genotypes.

### Mouse behavioral studies

Mice for behavioral studies were handled for 5 days before behavioral testing and habituated for at least 1 hour prior to experiments. Open field (OF) test, Novel object recognition (NOR) test, Morris water maze (MWM) test, visible platform test, and passive avoidance (PA) test were carried out as described [16, 17].

### Preparation of acute hippocampal slices and synaptic electrophysiology

Transverse hippocampal slices of 400 μm thick were prepared as described previously [18]. Slices were maintained at room temperature for at least 2 hours before experimentation in artificial cerebrospinal fluid (ACSF). For synaptic electrophysiology, slices were transferred to preheated (32° C) recording chambers where they were superfused with ACSF. High-frequency stimulation (HFS, consisting of two 1-sec 100 Hz trains separated by 60 sec) was delivered to induce late-LTP (L-LTP) [12].

### Western blots

Brain tissues were dissected, and flash frozen on dry ice. Protocols for Western blot were as described [12]. Resources of antibodies were as described [12].

### Surface sensing of translation (SUnSET) assay

Slices were maintained in ACS at room temperature for at least 2 hours before experiments. Slices were further incubated for 1 hour at 32° C in ACSF containing Puromycin (1 μg/mL). Micro-dissected area CA1 slices were used for Western blot analysis. Puromycin labeled proteins and *de novo* proteins synthesis were detected by using the anti-puromycin antibody, and quantified from the total lane (15 to 250 kDa).

### Transmission electron microscopy (TEM)

Protocols for TEM sample preparation and imaging were described in our recent studies [12].

### Mass spectrometry (MS)/ proteomic analysis

MS experiments were performed at Rutgers University center for advanced biotechnology and medicine using a Dionex rapid-separation liquid chromatography system interfaced with a QE HF (Thermo Fisher Scientific). Details for MS analysis were described previously [19].

### Statistical analysis

Data are presented as mean ± SEM. A two-tailed unpaired Student’s *t*-test was used for two groups comparisons. One-way ANOVA and post hoc tests (when applicable) were used to compare multiple groups. *p* < 0.05 were considered statistically significant. Sample size was based on previous publication [12]. GraphPad Prism software was applied for data analysis. Outliers were assessed by Grubbs test.

## Results and Discussion

### Genetic suppression of the neuronal AMPKα1 isoform improves aging-related impairments of recognition memory

**Figure 1:**
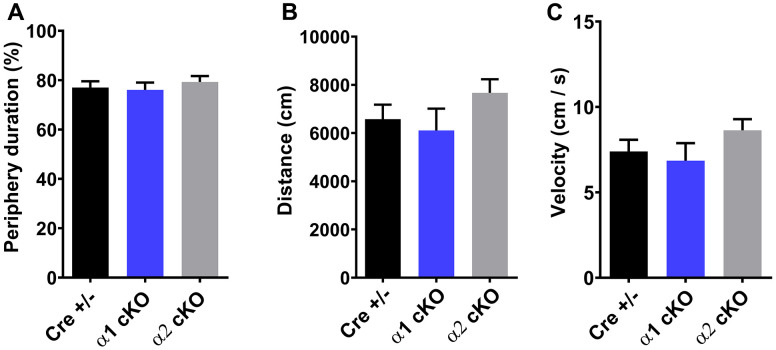
Open field test (OF) showed unaltered periphery duration (**A**), traveling distance (**B**), and traveling velocity (**C**) for both AMPKα1 cKO and AMPKα2 cKO mice. n=15 for Cre+/-, n=13 for AMPKα1 cKO, n=11 for AMPKα2 cKO. *p*>0.05. One-way ANOVA.

**Figure 2:**
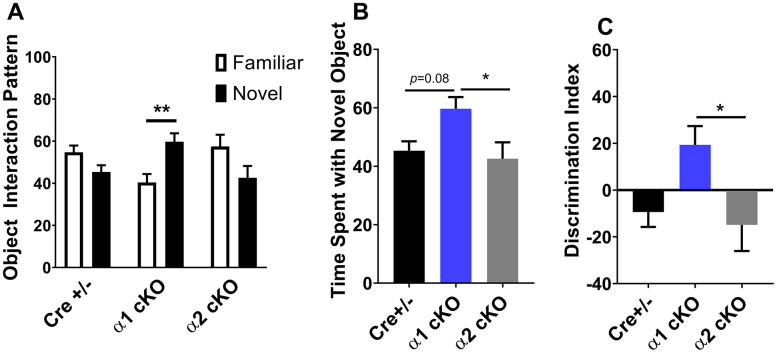
**Effects of AMPKα isoform reduction on recognition memory in aged mice.** (**A**) Novel object recognition (NOR) experiments demonstrated that long-term recognition memory was impaired with aging in Cre+/- and AMPKα2 cKO mice. In contrast, aged AMPKα1 cKO mice showed normal recognition of novel objects over familiar objects. n=8 for Cre+/- and AMPKα1 cKO, n=10 for AMPKα2 cKO. **p<0.01. Unpaired two-tailed t-test. (**B**) Time spent with the novel object during the NOR test. n=8 for Cre+/- and AMPKα1 cKO, n=10 for AMPKα2 cKO. *p<0.05. One-way ANOVA and Tukey’s test. (**C**) NOR discrimination index [(time spent exploring novel object − time spent exploring familiar object) / total exploration time]. n=8 for Cre+/- and AMPKα1 cKO, n=10 for AMPKα2 cKO. *p<0.05. One-way ANOVA and Tukey’s test.

We developed the AMPKα1/α2 cKO mice as described. In these transgenic mice, expression of either the α1 or the α2 isoform is conditionally reduced in excitatory neurons [12]. The mice were aged to 19-22 months old, when multiple aspects of cognition decline in wild-type (WT) mice [2]. We performed a series of behavioral tasks to assess various cognitive functions in the aged AMPKα1 cKO and α2 cKO mice, with the Cre+/- mice as the control. In the open field (OF) test, we did not observe difference among all the three groups of mice in the measurement of periphery duration and travel distance or velocity, indicating unaltered locomotor activities and anxiety levels with suppression of either AMPKα isoforms (Figure 1A–1C). We next performed the novel object recognition (NOR) task to evaluate their long-term recognition memory (24 hours interval between the exploration day and testing day) [20]. In agreement with our previous studies [2], aged Cre+/- mice exhibited impaired recognition memory, as they did not show a preference for the novel object measured by object interactions (Figure 2A left). Notably, aged AMPKα1 cKO mice interacted more with novel than with the familiar objects (*p*<0.01), indicating normal recognition memory (Figure 2A middle). In comparison, aged AMPKα2 cKO mice displayed a deficiency in recognition memory (Figure 2A right). Interestingly, there is a trend that the aged AMPKα2 cKO mice spent more time with the familiar object (*p*=0.07). Results from the analysis of the interactions with the novel objects showed that the long-term recognition memory was improved in the aged AMPKα1 cKO mice compared to the AMPKα2 cKO mice (*p*<0.05) (Figure 2B). There was also a trending improvement of recognition memory in the aged AMPKα1 cKO mice compared to the control mice (p=0.08) (Figure 2B). We further analyzed the discrimination index (calculated as time spent with novel object – familiar object) /total interaction time) based on the NOR performance, and confirmed that the aged AMPKα1 cKO mice exhibited improved recognition memory (positive high index), compared to aging-related impaired cognition in the other two groups (Figure 2C).

**Figure 3:**
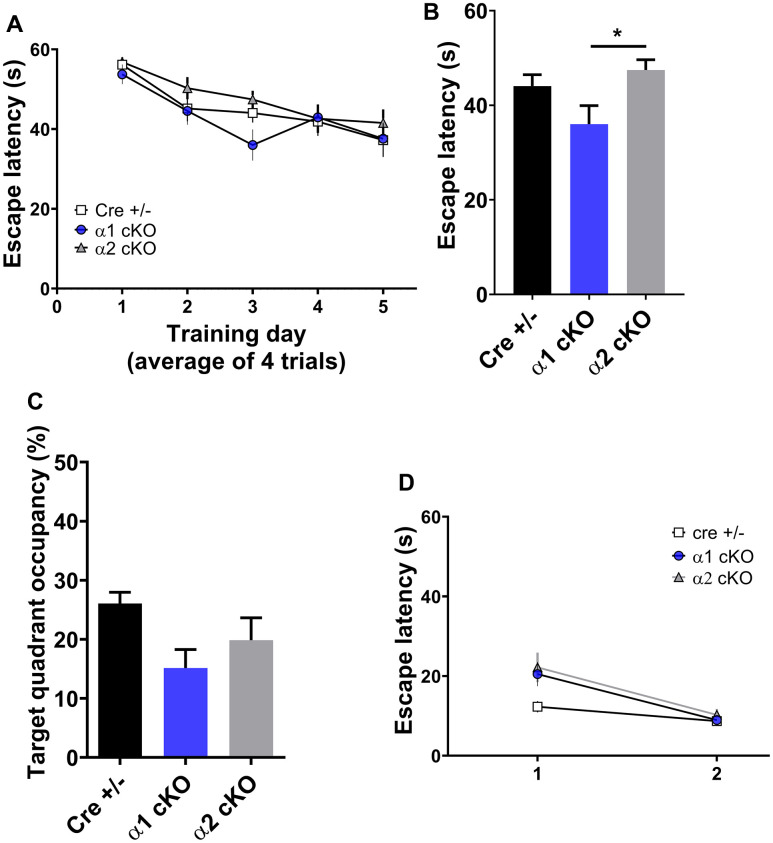
**Effects of AMPKα isoform reduction on spatial learning and memory in aged mice.** (**A**) The training phase of the hidden platform Morris water maze (MWM) test showed day-to-day escape latency of the mice. n=14 for Cre+/-, n=15 for AMPKα1 cKO and AMPKα2 cKO. *p*=0.78 (interaction between groups). Two-way ANOVA. (**B**) Escape latency on day 3 of the MWM test. *p<0.05. One-way ANOVA and Tukey’s test. (**C**) Measurement of the target quadrant occupancy in the probe trial phase of the MWM test, n=14 for Cre+/-, n=15 for AMPKα1 cKO and AMPKα2 cKO. *p*=0.056, One-way ANOVA. (**D**) Visible platform test. n=11-15 mice per group. *p*>0.05 One-way ANOVA.

Next, we examined the spatial learning/ memory of the mice with the classic Morris water maze (MWM) assay. Consistent with our recent report [2], aged Cre+/- mice exhibited defects in spatial learning and memory indicated by insignificant day-to-day decrease of escapes latency during the learning/training stage (Figure 3A). During the probe trial test, the aged Cre+/- mice also showed reduced target quadrant occupancy and “platform” crossing (Figure 3C and Supplementary Figure 1D). Unlike the results from the NOR test, both the AMPKα1 cKO and α2 cKO mice displayed similar spatial learning/memory impairments during the MWM test compared to the performance of the control group (Figure 3A, 3C and Supplementary Figure 1A, 1D). Further analysis revealed that the performance of the AMPKα1 cKO mice on day 3 of the MWM training phase was better than that of the AMPKα2 cKO mice (Figure 3B). In addition, we did not observe differences in swimming distance or velocity during the probe trial test among the three groups of mice (Supplementary Figure 1B, 1C). To evaluate the effects of AMPKα isoform suppression on other non-cognitive factors (e.g. vision, motivation, and swimming ability), we performed a visible platform task in which the escape platform is labeled with a flag and moved between trials [12]. We found that the escape latency during the visible platform test was unaltered in day 2 among all groups (Figure 3D). Interestingly, the Cre+/- mice exhibited shorter escape latency compared to the AMPKα2 cKO mice in day 1 (*p*<0.05) (Figure 3D).

Additionally, we investigated the effects of AMPKα isoform suppression on the performance of fear-associated learning and memory using the passive avoidance (PA) behavioral task. In the PA task, the mice learn to avoid an environment (measured by the entry latency on the testing day), where they had been shocked a day before (training day). The performance of all three groups of mice was similar during the training day. For the testing day, however, the aged AMPKα1 cKO mice showed a trend of improved fear learning/memory compared to the aged Cre+/- group (*p*=0.06). Moreover, there is no difference between the AMPKα2 cKO mice and the control group (Supplementary Figure 2). Combined with the results from the NOR and MWM tests, inhibition of the neuronal AMPKα1 isoform (but not the AMPKα2 isoform) can alleviate aging-related impairments of object recognition meanwhile does not improve spatial learning and memory deficits in aged mice. It is known that both the NOR and MWM tests are hippocampal-dependent. However, other brain regions such as prefrontal cortex might play distinct roles in these behavioral tasks [21, 22]. It would be intriguing for future studies to elucidate potential brain-region- or cell type-roles of AMPKα isoforms in aging-related regulation of cognitive function.

### Genetic suppression of the neuronal AMPKα1 isoform does not alter hippocampal long-term synaptic plasticity in aged mice

**Figure 4:**
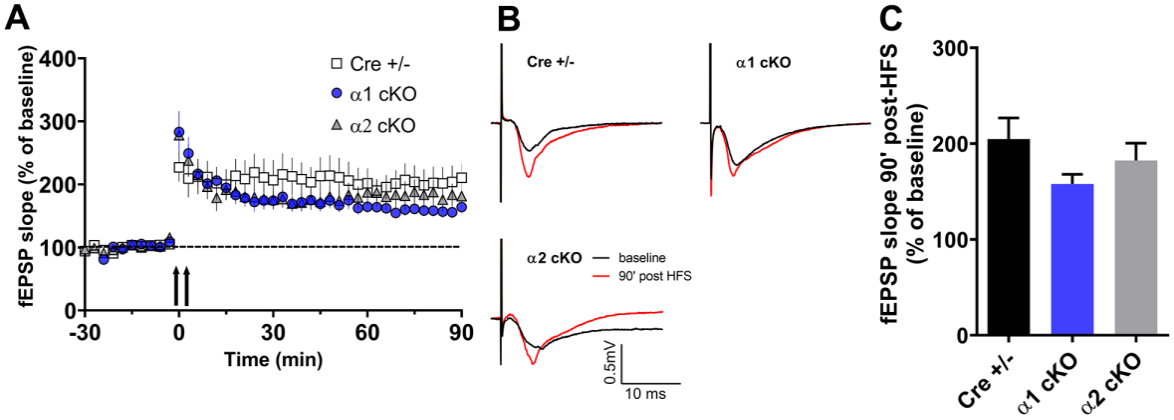
**Effects of AMPKα isoform reduction on hippocampal long-term synaptic plasticity in aged mice.** (**A**) Hippocampal late LTP (L-LTP) induced by two-train high-frequency stimulation (HFS, denoted by the arrows). (**B**) Representative fEPSP traces before and 90 minutes after HFS. (**C**) Cumulative data showing quantification of mean fEPSP slopes 90 min after delivery of HFS. n=13 for Cre+/- and AMPKα2 cKO, n=10 for AMPKα1 cKO. *p*=0.23, One-way ANOVA.

Furthermore, we examined whether suppression of the AMPKα isoforms affects hippocampal long-term potentiation (LTP), a form of synaptic plasticity that is considered as a cellular model for learning and memory [23]. In this study, we induced protein synthesis-dependent late LTP by applying a strong electric stimulation protocol [2]. The LTP in the aged Cre+/- mice appeared to be normal (Figure 4), which is consistent with our previous findings in aged wild mice [2]. In addition, the AMPKα1 cKO and α2 cKO mice showed similar hippocampal LTP compared to the Cre+/- mice (Figure 4).

### Phosphorylation levels of hippocampal eIF2α in aged mice are reduced by suppression of the neuronal AMPKα1 isoform in aged mice

**Figure 5:**
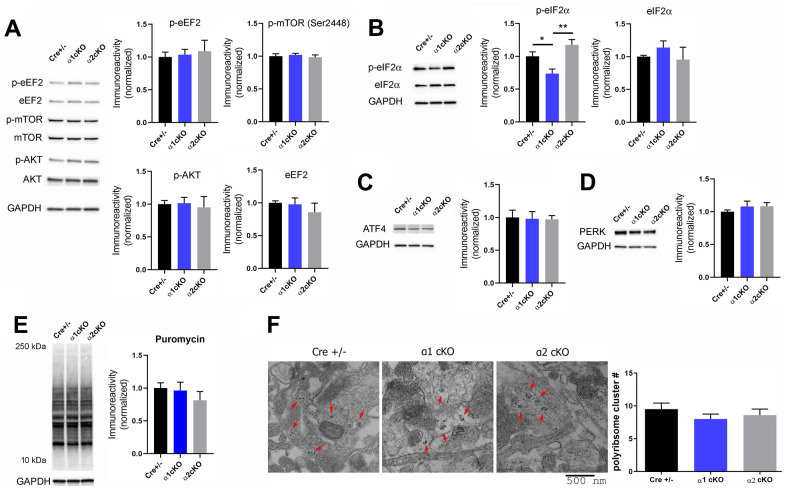
**Investigation of signaling pathways associated with AMPKα isoform inhibition in aged mice revealed de-phosphorylation of eIF2α in the hippocampus of the AMPKα1 cKO mice.** (**A**) Western blot experiments showed no alterations in phosphorylation levels of eEF2 (Thr56), mTOR (Ser2448), and AKT (Ser473) in hippocampal synaptosome lysate of AMPKα1 cKO or AMPKα2 cKO mice, compared to the Cre+/- mice. Representative Western blot gels and quantification data presented in bar graphs are shown. n= 4-6 per group. *p*>0.05, One-way ANOVA. (**B**) Levels of phospho-eIF2α (Ser51) were decreased in hippocampal synaptosome lysate of AMPKα1 cKO mice, compared to Cre+/- or AMPKα2 cKO mice group. n=7-8 per group. *p<0.05, **p<0.01, One-way ANOVA and Tukey’s test. (**C**, **D**) Levels of ATF4 and PERK were not altered in either AMPKα1 cKO or AMPKα2 cKO mice. n=5-6 per group. *p*>0.05, One-way ANOVA. (**E**) Representative images and quantification from the SUnSET de novo protein synthesis assay. n=3-4. *p*>0.05, One-way ANOVA. (**F**) Hippocampal polyribosome formation was unaltered in AMPKα1 cKO or AMPKα2 cKO mice, compared to the Cre+/- group. Representative transmission electron microscopy (TEM) images of hippocampal CA1 and cumulative data of polyribosome quantification were shown. Polyribosomes were indicated with red arrows. n=3 mice per group (8-10 ROI measurements per mouse). *p*=0.43. One-way ANOVA.

We seek to understand molecular mechanisms underlying the behavioral phenotypes associated with the suppression of AMPKα isoforms in aged mice. It has been reported that inhibition of overall AMPK activity results in phosphorylation of eEF2 and/or activation of the mTORC1 signaling, which would increase general protein synthesis [11]. Interestingly, we did not observe alterations of eEF2 phosphorylation in the hippocampus of either the AMPKα1 cKO or α2 cKO mice compared to the control group (Figure 5A). In addition, suppression of either AMPKα isoform did not affect the mTORC1 signaling in the hippocampus of the aged mice, as assessed by phosphorylation levels of mTOR (at both Ser2448 and Ser2481 sites), and of the two downstream substrates: p70S6K and 4EBP (Figure 5A and Supplementary Figure 3). Consistently, we also did not observe any significant effects of AMPKα isoform suppression on the phosphorylation levels of AKT, a well-established upstream regulator of mTORC1 (Figure 5A).

We recently reported, in young mice, that reduction of AMPKα2 results in increased phosphorylation of the eIF2α, which is associated with inhibition of translational capacity [12]. In contrast, here we did not observe alterations of eIF2α phosphorylation in the hippocampus of the aged AMPKα2 cKO mice compared to the Cre+/- mice group (Figure 5B). Notably, phosphorylation levels of eIF2α in the hippocampus of the aged AMPKα1 cKO mice were significantly decreased compared to those in either the control or the AMPKα2 cKO mice (Figure 5B). We further investigated ATF4, whose expression might be associated with eIF2α phosphorylation [24, 25]. We did not observe any changes in the protein levels of hippocampal ATF4 in AMPKα1 or α2 cKO mice (Figure 5C). Additionally, protein levels of eIF2α kinase PERK were also unaltered among all the groups (Figure 5D).

Phosphorylation of eIF2α is considered to result in repression of general protein synthesis [26]. We thus examined de novo protein synthesis in living hippocampal slices by the SUnSET assay [16]. Surprisingly, the results indicate no change of general protein synthesis levels (assessed by puromycin incorporation) among all three groups (Figure 5E). We further carried out transmission electron microscopy experiments to investigate regulation of hippocampal dendritic polyribosomes, an indicator of new protein synthesis [27]. We found that the presence of polyribosomes was unaffected by the suppression of either AMPKα1 or α2, compared to the control group (Figure 5F). Potential mechanisms for the unaltered overall de novo protein synthesis (in consideration of the eIF2α phosphorylation regulation) may involve the regulation of the protein degradation process and a balance between both upregulation and downregulation of individual protein synthesis (see the proteomics results below). Taken together, suppression of the AMPKα isoform in neurons does not alter the general mRNA translation rate in the hippocampus of aged mice.

### Proteomic analysis reveals distinct alterations of protein expression levels associated with suppression of the neuronal AMPKα isoform in aged mice

**Figure 6:**
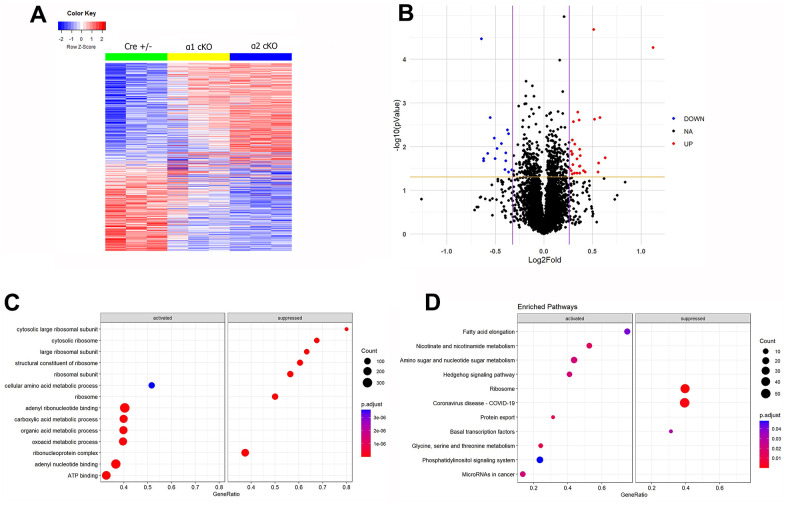
**Mass spectrometry (MS)-based proteomics analysis reveals distinct alterations of protein expression levels associated with suppression of the neuronal AMPKα isoform in aged mice.** (**A**) A heat map generated from the MS proteomics data showed the differentially newly synthesized proteins (667 proteins) across the three experimental groups. (**B**) A volcano plot showed the fold changes of protein expression in AMPKα2 cKO vs AMPKα1 cKO mice. Red dots represent those significantly upregulated proteins (29 proteins). Blue dots represent those significantly downregulated proteins (16 proteins). Black dots represent the proteins whose expression levels were not significantly different between the AMPKα1 cKO and AMPKα2 cKO mice. (**C**) Gene Ontology (GO) analysis of the differentially regulated proteins in AMPKα2 cKO vs AMPKα1 cKO mice. (**D**) Gene Set Enrichment Analysis (GSEA) of differentially regulated proteins in AMPKα2 cKO vs AMPKα1 cKO mice. All proteomics analysis was performed with the R program. Heat map and volcano plot were generated with ggplot2 package (version 3.3.5) in R (version 4.1.2). GO analysis and GSEA were done with clusterProfiler package (version 4.2.2) in R (version 4.1.2).

To gain insights into the potential regulation of the expression of individual proteins associated with the AMPKα isoform reduction, we performed mass spectrometry (MS)-based proteomic experiments. (Figure 6C, 6D). Most significantly changed hippocampal proteins (up-regulated or down-regulated) in the AMPKα1 cKO or α2 cKO mice compared to the Cre+/- mice were shown in Tables 1–4 (illustrated in Supplementary Figure 4). To understand the differences in proteomics between AMPKα1 cKO and AMPKα2 cKO mice, we further generated a “volcano” plot to show the fold changes of protein expression levels in the AMPKα2 cKO mice compared to those in the AMPKα1 cKO mice (Figure 6B). In brief, levels of 29 proteins were increased and 16 proteins were decreased in the AMPKα2 cKO mice compared to the AMPKα1 cKO mice. Detailed lists of these proteins were shown in Tables 5, 6. To further understand how these regulated proteins are involved in different biological processes, we carried out the Gene Ontology (GO) analysis and the Gene Set Enrichment Analysis (GSEA) in AMPKα2 cKO and AMPKα1 cKO mice (Figure 6C, 6D). GO analysis of the differentially regulated proteins between AMPKα1 cKO and AMPKα2 cKO mice suggested that expression of proteins involved in the biological processes related to ribosome subunit biology was suppressed in the AMPKα2 cKO mice compared to AMPKα1 cKO mice. In contrast, expression of proteins involved in the metabolism-related processes was activated in the AMPKα2 cKO mice compared to AMPKα1 cKO mice (Figure 6C). Consistent with GO analysis, GSEA analysis shows that ribosome-related pathways were suppressed, while metabolism-related pathways were activated in AMPKα2 cKO mice compared to AMPKα1 cKO mice (Figure 6D). Interestingly, we found COVID-19-related pathways were suppressed in the AMPKα2 cKO mice, suggesting AMPKα isoforms may play distinct role in viral infection (Figure 6D).

**Table 1:** Top 10 down-regulated proteins in AMPKα1 cKO mice (compared to Cre+/-).

**Protein names**	**Gene names**	**P value**	**Log2Fold**
LIM domain and actin-binding protein 1	Lima1	0.013357	-1.02045
Interleukin-34	Il34	0.017373	-0.52683
Peptidyl-prolyl cis-trans isomerase E	Ppie	0.011286	-0.5126
Gamma-aminobutyric acid receptor subunit alpha-4	Gabra4	0.0333	-0.48554
Guanine nucleotide binding protein, beta 2	Gnb2	0.009171	-0.46737
Trafficking protein, kinesin binding 2	Trak2	0.00616	-0.42482
Beta-hexosaminidase subunit alpha	Hexa	0.016773	-0.42092
Cathepsin L1	Ctsl	0.014357	-0.36424
Prospero homeobox protein 1	Prox1	0.003035	-0.33767
Transmembrane emp24 domain-containing protein 5	Tmed5	0.025036	-0.33653

**Table 2:** Top 10 up-regulated proteins in AMPKα1 cKO mice (compared to Cre+/-).

**Protein names**	**Gene names**	**P value**	**Log2Fold**
5-AMP-activated protein kinase subunit beta-1	Prkab1	0.019052	0.423145
GTP-binding protein Rhes	Rasd2	0.034253	0.437368
Tropomyosin alpha-1 chain	Tpm1	0.021857	0.448768
Dynein light chain 2, cytoplasmic	Dynll2	0.045608	0.468517
Palmitoyltransferase	Zdhhc8	0.015551	0.523234
Ubiquitin-conjugating enzyme E2 D1	Ube2d1	0.0454	0.578678
Short transient receptor potential channel 6	Trpc6	0.006422	0.607953
Progressive ankylosis protein	Ank	0.00454	0.803404
FAD-dependent oxidoreductase domain-containing protein 2	Foxred2	0.002152	0.919643
Nuclear factor NF-kappa-B p100 subunit	Nfkb2	0.00424	2.891302

**Table 3:** Top 10 down-regulated proteins in in AMPKα2 cKO mice (compared to Cre+/-).

**Protein names**	**Gene names**	**P value**	**Log2Fold**
NAD(P) transhydrogenase, mitochondrial	Nnt	0.00093	-1.12257
LIM domain and actin-binding protein 1	Lima1	0.011128	-1.05596
U1 small nuclear ribonucleoprotein C	Snrpc	0.030598	-0.88195
Centrosomal protein of 290 kDa	Cep290	0.03503	-0.85265
Peroxiredoxin 5	Prdx5	0.004656	-0.7859
Serine protease inhibitor A3B	Serpina3b	0.001913	-0.76149
Tubulin alpha chain-like 3	Tubal3	0.012086	-0.73929
U6 snRNA-associated Sm-like protein LSm7	Lsm7	0.031444	-0.72817
Transmembrane emp24 domain-containing protein 5	Tmed5	0.002663	-0.69864
N6-adenosine-methyltransferase subunit METTL14	Mettl14	0.046065	-0.65453

**Table 4:** Top 10 up-regulated proteins in in AMPKα2 cKO mice (compared to Cre+/-).

**Protein names**	**Gene names**	**P value**	**Log2Fold**
Coiled-coil domain-containing protein 97	Ccdc97	0.000594	0.833568
Transmembrane protein 69	Tmem69	0.022074	0.959761
Putative hydroxypyruvate isomerase	Hyi	0.000394	0.971629
Histone deacetylase 3	Hdac3	0.002509	0.982715
Myosin-10	Myh10	0.003434	0.996647
Putative tyrosine-protein phosphatase auxilin	Dnajc6	0.014514	1.097624
Progressive ankylosis protein	Ank	0.000313	1.211377
Internexin neuronal intermediate filament protein, alpha	Ina	0.006883	1.231642
Dedicator of cytokinesis protein 6	Dock6	0.006143	1.297596
Nuclear factor NF-kappa-B p100 subunit	Nfkb2	0.000778	3.381316

**Table 5:** Up-regulated proteins in AMPKα2 cKO mice compared to AMPKα1 cKO mice.

**Protein names**	**Gene names**	**P value**	**Log2Fold**
Putative hydroxypyruvate isomerase	Hyi	<0.0001	1.13
propionyl-CoA carboxylase subunit beta	Pccb	0.0182	0.63
Ribonuclease P protein subunit p14	Rpp14	0.0022	0.58
Histone deacetylase 3	Hdac3	0.0239	0.56
Dedicator of cytokinesis protein 6	Dock6	0.0384	0.56
Haloacid dehalogenase-like hydrolase domain-containing protein 3	Hdhd3	0.0024	0.52
Insulin-degrading enzyme	Ide	<0.0001	0.51
Methyl-CpG-binding domain protein 2	Mbd2	0.0382	0.43
Adiponectin receptor protein	Adipor	0.0356	0.41
Delta-aminolevulinic acid dehydratase	Alad	0.0163	0.38
Complement C4 beta chain	C4b	0.0280	0.37
Activity-regulated cytoskeleton-associated protein	Arc	0.0116	0.37
ETS domain-containing transcription factor	Erf	0.0406	0.37
Vasculin	Gpbp1	0.0283	0.37
Probable proline--tRNA ligase, mitochondrial	Pars2	0.0025	0.36
Glycogen synthase, muscle	Gys1	0.0016	0.35
1,2-dihydroxy-3-keto-5-methylthiopentene dioxygenase	Adi1	0.0192	0.34
Dual specificity testis-specific protein kinase 1	Tesk1	0.0405	0.34
Transmembrane protein 168	Tmem168	0.0088	0.32
trafficking protein, kinesin binding 2	Trak2	0.0410	0.31
Hydroxypyruvate isomerase	Hyi	0.0027	0.30
Glutamate-rich WD repeat-containing protein 1	Grwd1	0.0263	0.30
T-box brain protein 1	Tbr1	0.0424	0.29
Oligoribonuclease, mitochondrial	Rexo2	0.0071	0.29
Probable ATP-dependent RNA helicase DDX58	Ddx58	0.0147	0.29
Uncharacterized protein CXorf38 homolog	1810030O07Rik	0.0153	0.29
Sodium bicarbonate cotransporter 3	Slc4a7	0.0360	0.28
Cyclin-L2	Ccnl2	0.0129	0.28
Cytosolic endo-beta-N-acetylglucosaminidase	Engase	0.0318	0.27

**Table 6:** Down-regulated proteins in AMPKα2 cKO mice compared to AMPKα1 cKO mice.

**Protein names**	**Gene names**	**P value**	**Log2Fold**
5-AMP-activated protein kinase catalytic subunit alpha-2	Prkaa2	<0.0001	-0.64
Calmodulin-binding transcription activator 2	Camta2	0.0214	-0.62
WD repeat and FYVE domain containing 1	Wdfy1	0.0191	-0.62
Aminoacylase-1	Acy1	0.0145	-0.58
Protein FAM177A1	Fam177a1	0.0021	-0.55
Disks large-associated protein 1	Dlgap1	0.0064	-0.51
Necdin	Ndn	0.0189	-0.50
UDP-N-acetylglucosamine transferase subunit ALG14 homolog	Alg14	0.0111	-0.48
FYVE, RhoGEF and PH domain-containing protein 5	Fgd5	0.0086	-0.44
RNA binding protein fox-1 homolog 1	Rbfox1	0.0340	-0.40
Codanin-1	Cdan1	0.0213	-0.39
Bone morphogenetic protein receptor type-1B	Bmpr1b	0.0139	-0.39
3-ketoacyl-CoA thiolase A, peroxisomal	Acaa1a	0.0042	-0.38
Vesicle-associated membrane protein 4	Vamp4	0.0384	-0.36
Transmembrane emp24 domain-containing protein 5	Tmed5	0.0051	-0.36
Ig heavy chain V	Ighv	0.0342	-0.33

## CONCLUSIONS

In summary, the current study reported that suppression of neuronal AMPKα1 isoform can improve aging-related impairments of long-term recognition memory. Together with our previous studies in characterizing the young AMPKα transgenic mice, these novel findings point to a previously unrecognized role of AMPKα isoform homeostasis in cognition during development. The study indicates that the aging process might have distinct impact on the signaling pathways associated with the AMPKα isoforms, and future studies are necessary to determine the underlying mechanisms. Finally, it is appealing for future studies to explore whether targeting AMPKα isoform regulation could be a feasible strategy to mitigate aging-related cognitive impairments.

## Supplementary Material

Supplementary Figures
