# Supplementary material for: Isoform-specific effects of neuronal repression of the AMPK catalytic subunit on cognitive function in aged mice
Source: Aging (Albany NY). 2023 Feb 26;15(4):932–46. doi: 10.18632/aging.204554 (PMC10008489; doi:10.18632/aging.204554)
Supplement: Supplementary Figures [file aging-15-204554-s001.pdf]

SUPPLEMENTARY FIGURES

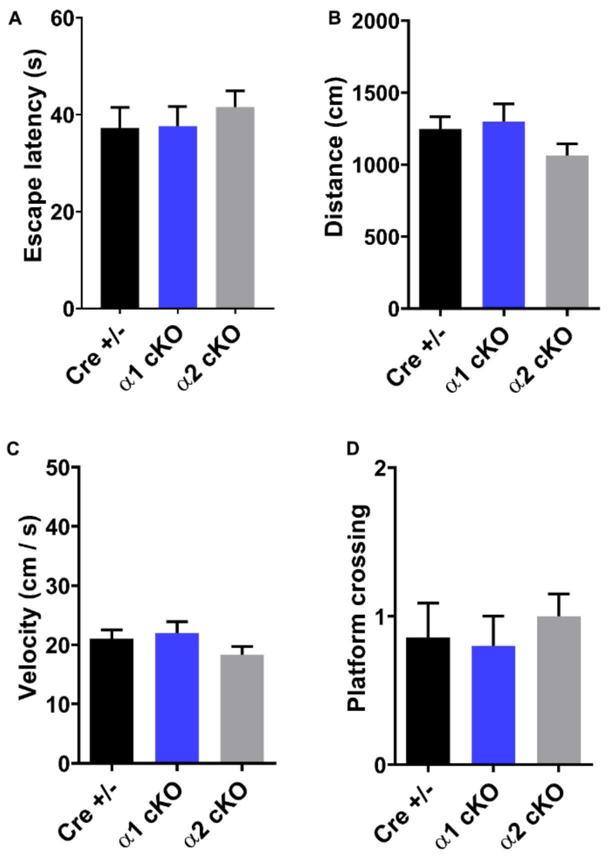

Supplementary Figure 1. Characterization of behavioral phenotypes in aged transgenic mice with AMPKα isoform suppression. (**A**) Escape latency on day 5 of the MWM test. (**B**) Swimming distance during the probe trial of the MWM test. (**C**) Swimming velocity during the MWM probe trial. (**D**) Measurement of "platform" crossing in the probe trial of the MWM test. , n=14 for Cre+/-, n=15 for AMPKα1 cKO and AMPKα2 cKO. *p*=0.22, One-way ANOVA.

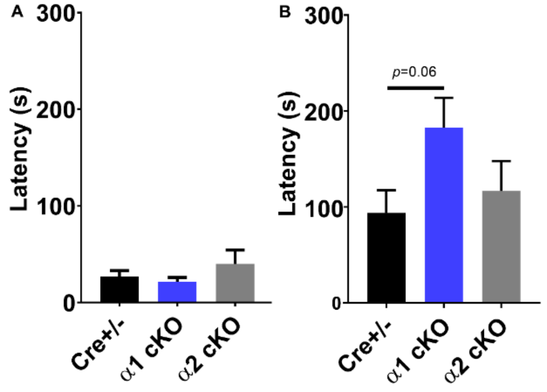

Supplementary Figure 2. Effects of AMPKα isoform reduction on fear-associated memory in aged mice. (**A**) Training day of the passive avoidance (PA) test. (**B**)Testing Day of the PA task. n=18 for Cre+/- and AMPKα1 cKO, n=13 for AMPKα2 cKO. One-way ANOVA and Tukey's test.

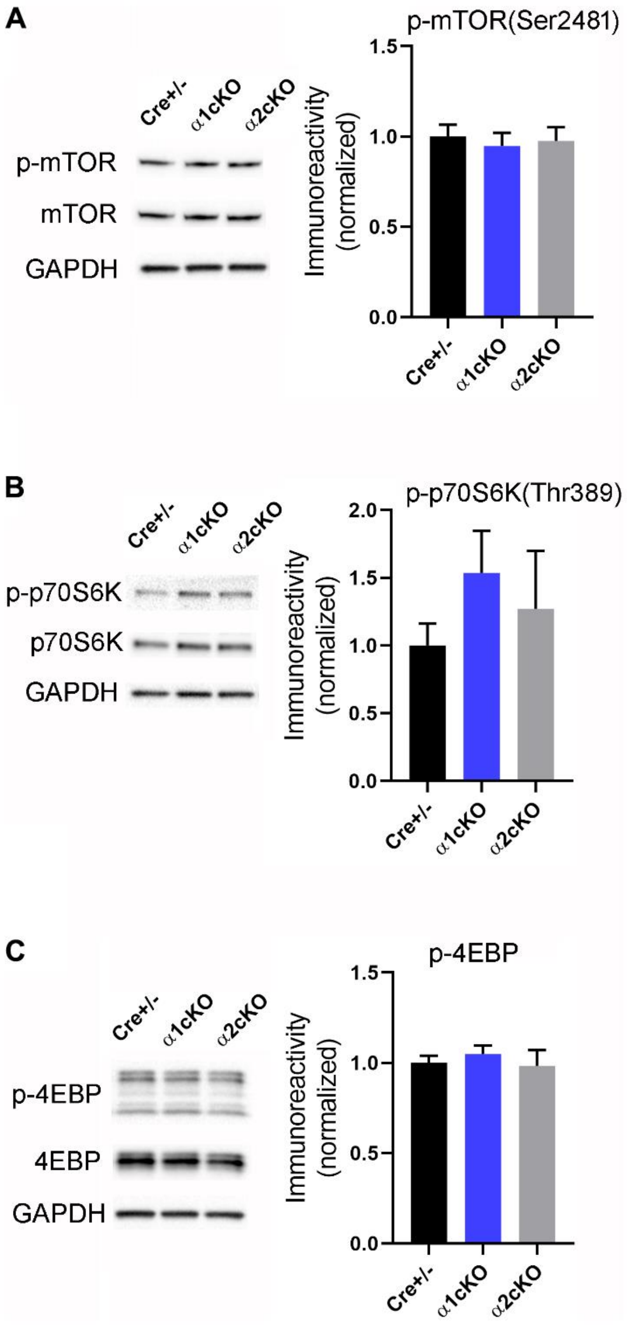

Supplementary Figure 3. Examination of the mTORC1 signaling pathway in the hippocampus of the transgenic mice with AMPKα isoform suppression. Western blot experiments showed no alterations in phosphorylation levels of (**A**) mTOR (Ser2481), (**B**) p70S6K (Thr389), and (**C**) 4EBP. Representative Western blot gels and quantification data presented in bar graphs are shown. n=5-6 per group. p>0.05 One-way ANOVA.

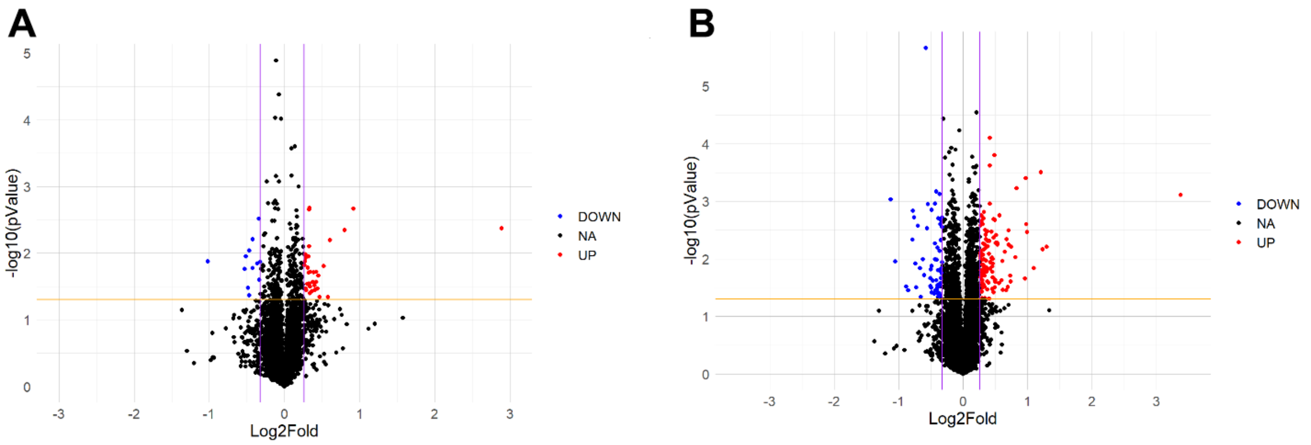

Supplementary Figure 4. Volcano plot showed the fold changes of protein expression in AMPKα1 cKO vs. Cre+/- (**A**) and AMPKα2 cKO vs. Cre+/- mice (**B**). Red dots represent those significantly upregulated proteins. Blue dots represent those significantly downregulated proteins. Black dots represent the proteins whose expression levels were not significantly altered.
